# Simultaneous Carpal Tunnel Syndrome and Guyon’s Canal Syndrome Caused by a Lipoma Arising From the Flexor Tenosynovium: A Case Report

**DOI:** 10.7759/cureus.97379

**Published:** 2025-11-20

**Authors:** Kazuhiro Ikeda, Hiromitsu Tsuge, Shotaro Teruya, Ryunosuke Watanabe, Shinzo Onishi

**Affiliations:** 1 Department of Orthopedic Surgery, Institute of Medicine, University of Tsukuba, Tsukuba, JPN; 2 Department of Orthopedic Surgery, Kikkoman General Hospital, Noda, JPN

**Keywords:** carpal tunnel syndrome, dorsal cutaneous branch of the ulnar nerve, guyon canal syndrome, lipoma, occult, tenosynovium, tumor

## Abstract

Carpal tunnel syndrome (CTS) is the most common peripheral entrapment neuropathy. While most cases are idiopathic, secondary CTS due to space-occupying lesions such as tumors is relatively uncommon. We report a rare case of simultaneous compression of the median nerve and the dorsal cutaneous branch of the ulnar nerve by a small lipoma arising from the flexor tenosynovium. Magnetic resonance imaging clearly demonstrated the lesion within the carpal tunnel and near Guyon’s canal. Surgical excision led to resolution of median nerve symptoms, whereas ulnar sensory disturbance persisted due to involvement of the unrecognized dorsal cutaneous branch of the ulnar nerve. The patient later developed cubital tunnel syndrome, and concurrent compression of the dorsal cutaneous branch of the ulnar nerve complicated neurological assessment. This case emphasizes the need for comprehensive assessment and early MRI evaluation in atypical or unilateral CTS.

## Introduction

Carpal tunnel syndrome (CTS) is the most frequent peripheral nerve entrapment, affecting approximately 3-5% of the general population [1]. As most cases are idiopathic, the diagnosis is usually established on the basis of physical examination and electrodiagnostic studies [2,3]. Therefore, imaging is not routinely required [4,5]. In contrast, approximately 3% of CTS cases are attributed to space-occupying lesions, including tumors, ganglia, anomalous muscles, and synovitis [6]. Although imaging studies are essential for diagnosing these lesions, their indication can be difficult to determine.

We encountered a case in which a lipoma arising from the flexor tenosynovium caused simultaneous compression of the median nerve and the dorsal cutaneous branch of the ulnar nerve (DCBUN). The lesion was clinically inconspicuous yet was clearly identified by magnetic resonance imaging (MRI), underscoring the importance of imaging in detecting such atypical causes of CTS. Moreover, the coexistence of multiple peripheral nerve symptoms posed a diagnostic challenge. These observations provide valuable clinical implications regarding the diagnostic evaluation of atypical CTS; therefore, we report this case.

## Case presentation

A 58-year-old woman presented with a history of several months of numbness in all five fingers and a palpable subcutaneous mass on the volar wrist. The palpable subcutaneous mass was found to be a cord-like structure along the course of the median nerve. Compression at this site elicited radiating pain to the index, middle, and radial aspects of the ring finger. In contrast, numbness in the ulnar digits (ring and little fingers) was mild. There was no apparent weakness in the intrinsic muscles supplied by either the median or the ulnar nerve. Side pinch strength, measured using a pinch gauge (R-364-B; Tiger Medical Instruments Co., Osaka, Japan), was 2.0 kg on the right and 5.0 kg on the left; this difference was presumed to be influenced by pain from preexisting carpometacarpal joint osteoarthritis.

**Figure 1:**
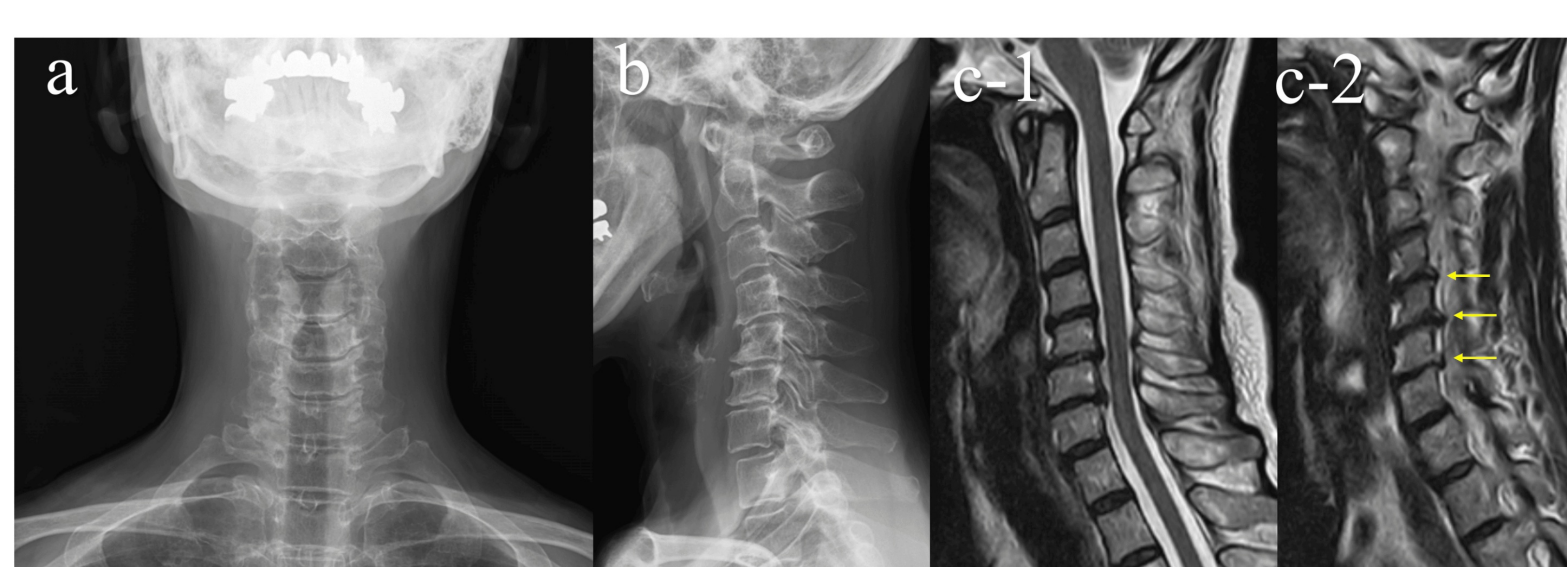
Cervical radiographs and MRI findings. (a) Anteroposterior radiograph showing a sloping-shoulder body habitus. (b) Lateral radiograph demonstrating localized cervical kyphosis. (c-1, c-2) Sagittal T2-weighted MRI showing facet joint osteoarthritis (yellow arrows) and disc bulging at C4/5, C5/6, and C6/7, without evidence of central canal stenosis. MRI, magnetic resonance imaging.

Spurling and Morey tests were negative. Cervical radiographs demonstrated localized kyphosis, and the patient had a sloping-shoulder body habitus. MRI revealed facet joint osteoarthritis and disc bulging at C4/5, C5/6, and C6/7, without evidence of central canal stenosis (Figure 1). Although these imaging findings indicated cervical or brachial plexus traction, physical examination findings did not suggest cervical radiculopathy or thoracic outlet syndrome.

**Figure 2:**
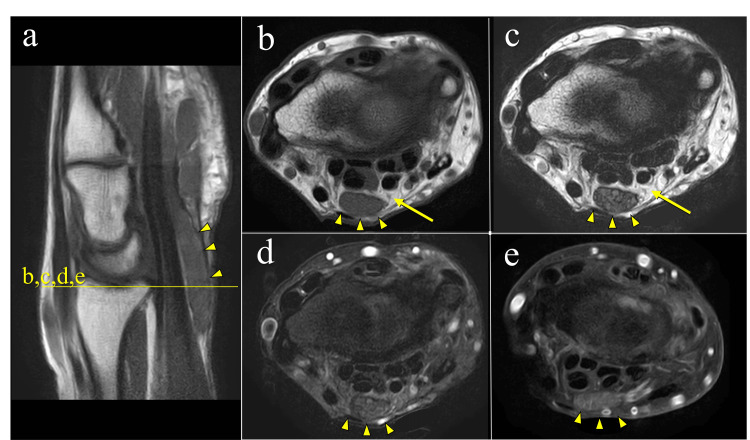
MRI findings at the initial visit. (a) T1-weighted sagittal image; (b) T1-weighted axial image; (c) T2-weighted axial image; (d) T2-SPIR transverse image. The median nerve was enlarged within the carpal tunnel, measuring 11.0 × 5.3 mm (cross-sectional area, 50 mm²; yellow arrowhead). The nerve showed homogeneous low signal intensity on both T1- and T2-weighted images, without contrast enhancement, consistent with a pseudo-neuroma. A well-defined mass was observed at the proximal carpal tunnel, showing high signal intensity on T1- and T2-weighted images, low signal intensity on SPIR, and demonstrating poor enhancement, findings consistent with a lipoma (yellow arrow). MRI, magnetic resonance imaging; SPIR, spectral presaturation with inversion recovery; TRA, transverse.

The Semmes-Weinstein monofilament test showed normal sensation (green) in all digits. However, nerve conduction studies revealed delayed terminal latency of the median nerve on the right (7.47 ms) compared with the left (5.01 ms). Ultrasonography demonstrated marked enlargement of the median nerve at the proximal level of the transverse carpal ligament, with a cross-sectional area of 50 mm² (reference value in patients with CTS: 12 mm² [7]), prompting further evaluation with MRI (Figure 2). MRI revealed a space-occupying lesion consistent with a lipoma at the proximal portion of the carpal tunnel, along with fusiform swelling of the median nerve suggestive of a pseudo-neuroma. Based on these findings, we diagnosed CTS secondary to a lipoma (Table 1).

**Table 1:** Preoperative evaluation of carpal tunnel syndrome. MMT, manual muscle testing; PT, pronator teres; FDS, flexor digitorum superficialis; APB, Abductor Pollicis Brevis; SW test, Semmes-Weinstein test; QuickDASH score, Quick Disability of the Arm, Shoulder, and Hand score.

Items	Findings
Physical test	Pharen test (-) Durkan test (+)
MMT (right/ left)	PT:5/5 FDS:5/5 APB:5/5
SW test (palm)	all green
Terminal latency of median nerve (right/ left)	7.47/ 5.01 msec
QuickDASH score (disability and pain)	34.1

Surgery

**Figure 3:**
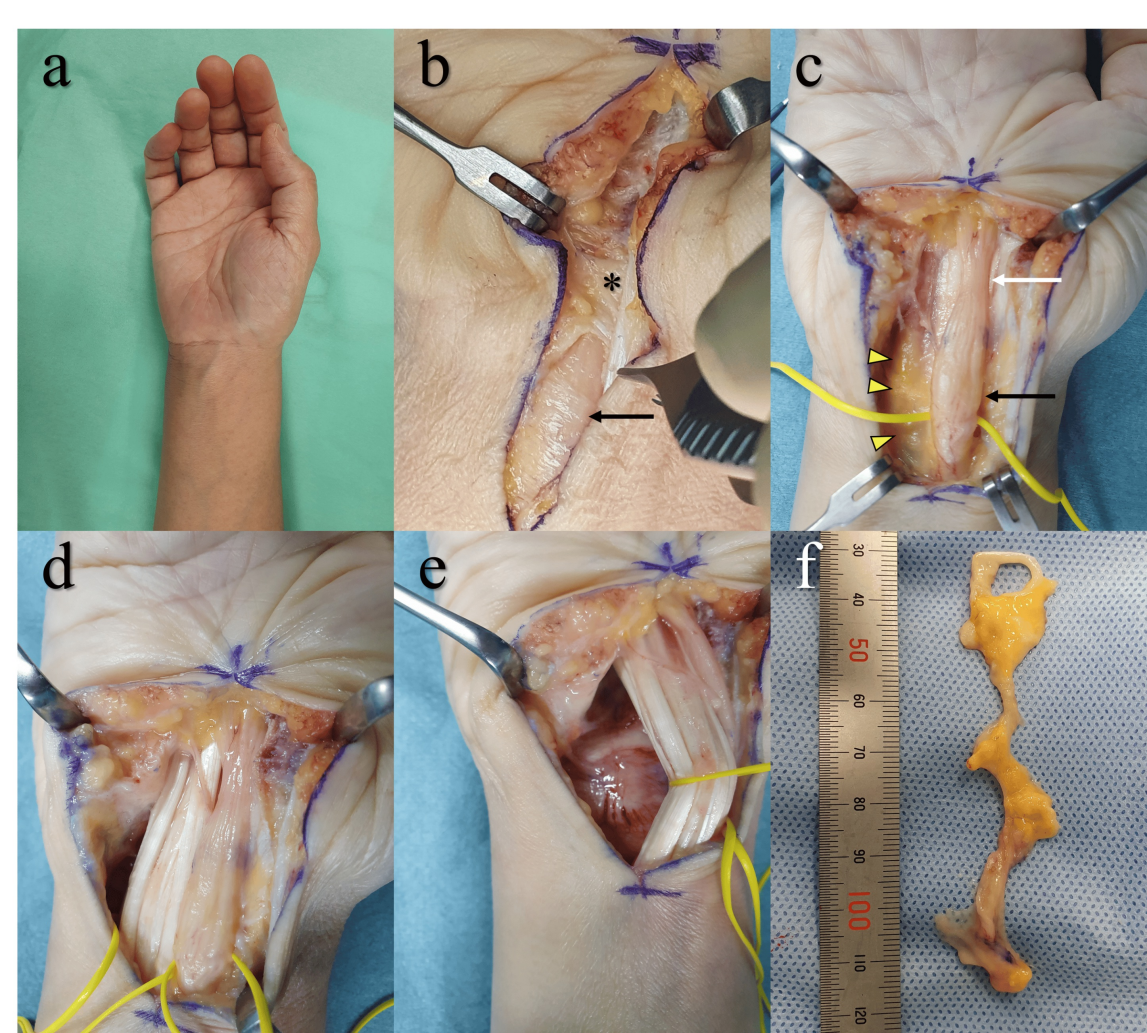
Intraoperative findings. (a) External appearance: no specific findings. (b) Before carpal tunnel release: the median nerve (black arrow) was enlarged proximal to the transverse carpal ligament (*). The ligament showed no apparent thickening. (c) After carpal tunnel release: the median nerve (black arrow) was enlarged at the proximal portion of the tunnel compared with the level within the tunnel (white arrow). A lipoma continuous with the flexor tenosynovium proliferated on the ulnar side of the enlarged median nerve. (d, e) After excision of the lipoma and flexor tenosynovium: there was no evidence of synovitis. (f) The excised specimen consisting of the lipoma and flexor tenosynovium.

**Figure 4:**
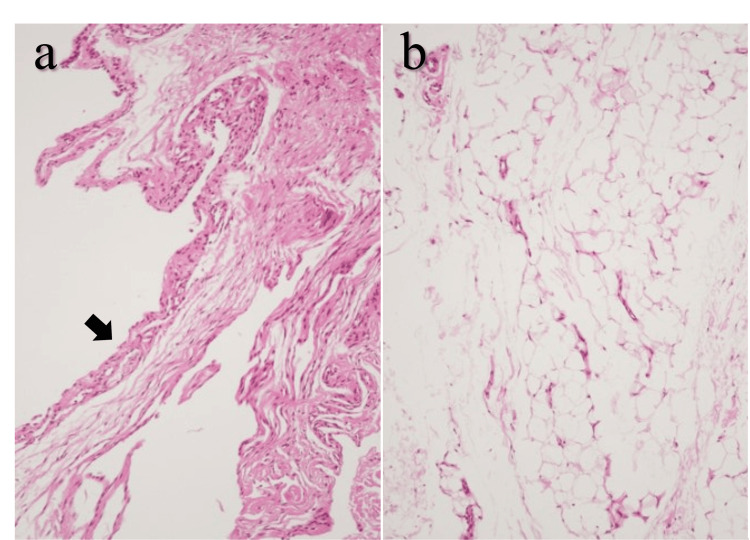
Histopathological findings. (a) Synovial tissue (×10). A single layer of synovial cells (arrow) covers the surface without evidence of active inflammation. (b) Lipoma (×40). Proliferation of mature adipocytes with small, uniform nuclei is observed, and no immature lipoblasts are present. These findings are consistent with a diagnosis of lipoma.

We released the carpal tunnel and excised the lipoma under general anesthesia (Figure 3). We made a zigzag incision from the proximal wrist region to the distal margin of the carpal tunnel. The transverse carpal ligament was not thickened, whereas the median nerve was enlarged at the proximal portion of the carpal tunnel. On the ulnar side of the median nerve, we identified a lipoma continuous with the flexor tenosynovium, which we excised completely and submitted for histopathological examination (Figure 4).

Postoperative course

Postoperative Month (POM) 1

Her subjective numbness in the median nerve distribution completely resolved.

POM6

The terminal latency of the median nerve had improved to 5.2 ms.

Postoperative Year (POY) 1

**Figure 5:**
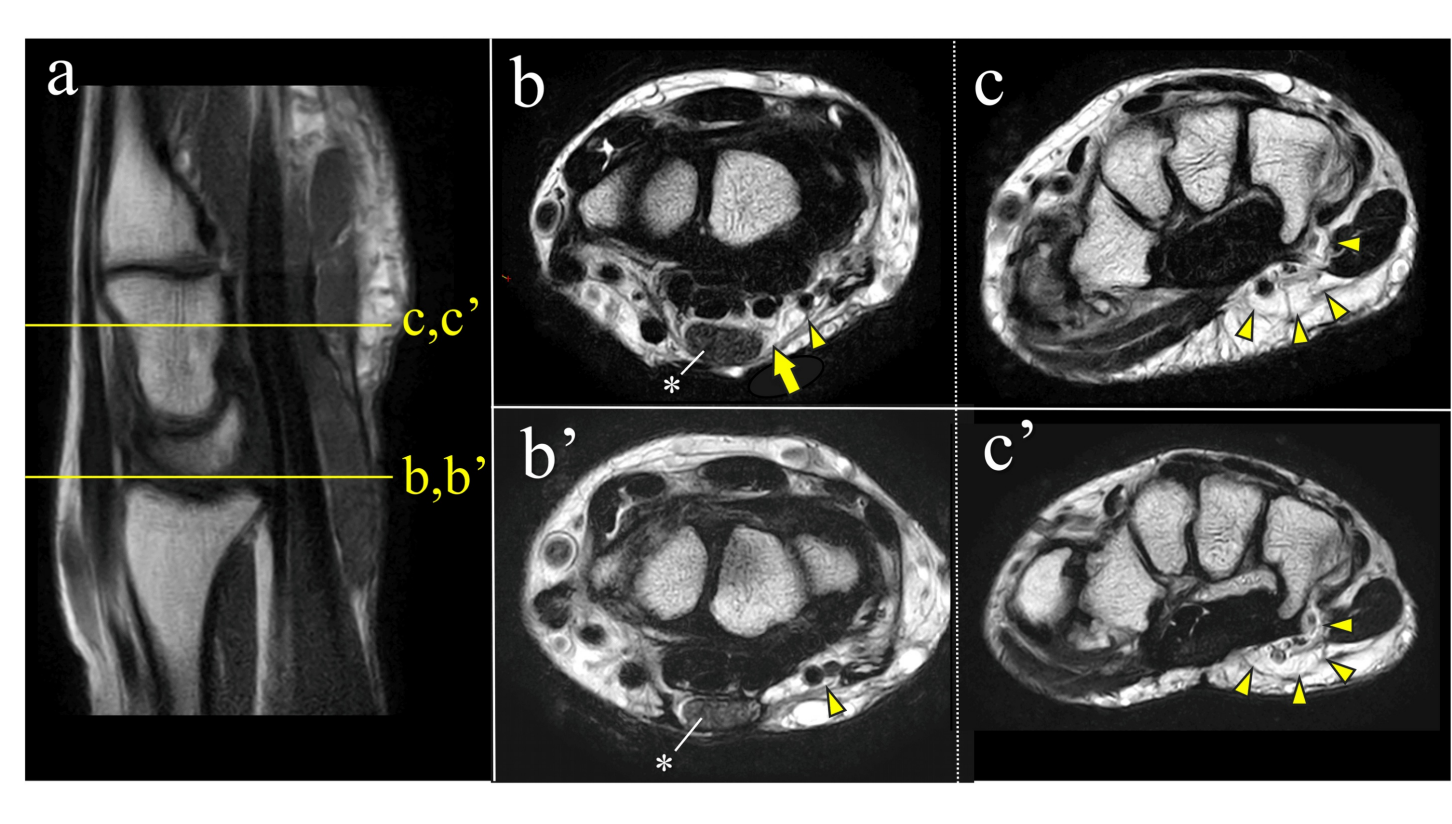
MRI findings of the lipoma at presurgery and POY1. (a) Scout view (T1-weighted sagittal image). (b, b′) T2-weighted sagittal images at the level of the proximal carpal and Guyon’s canals before surgery (b) and at POY1 (b′). The lipoma around the median nerve (b, yellow arrow) was excised and was no longer visible on the POY1 MRI (b′). The cross-sectional area of the median nerve decreased from 50 mm² preoperatively (b, *) to 35 mm² postoperatively (b’, *). A lipoma remained around the ulnar nerve proximally (b/b’, arrowhead). (c, c′) T2-weighted sagittal images distal to Guyon’s canal before surgery (c) and at POY1 (c′). A residual lipoma was evident distal to Guyon’s canal, around the ulnar nerve (yellow arrowheads). MRI, magnetic resonance imaging; POY, postoperative year.

The patient reported worsened numbness on the ulnar side of the right hand, which was more pronounced on the dorsal side compared with the palmar side. Tinel-like signs were elicited both proximal to the Guyon’s canal and at the cubital tunnel. Her numbness was particularly severe in the morning, and the elbow flexion test was positive. Muscle strength of the 3rd and 4th lumbricals and the abductor digiti quinti (ADQ) was reduced to manual muscle testing (MMT) grade 4. Nerve conduction studies of the ulnar nerve showed no delay in terminal latency, whereas the motor conduction velocity was slightly reduced across the cubital tunnel (Table 2). Postoperative MRI revealed a lipoma proximal to the Guyon’s canal. Review of the preoperative MRI showed that this lipoma was continuous with the one surrounding the median nerve (Figure 5). Based on these findings, the ulnar nerve symptoms were diagnosed as a combination of DCBUN compression caused by the lipoma and cubital tunnel syndrome. We proposed excision of the lipoma, neurolysis, and cubital tunnel release; however, the patient opted for conservative treatment because of work and caregiving responsibilities. Therefore, we fabricated a nighttime elbow extension splint to alleviate symptoms of cubital tunnel syndrome.

**Table 2:** Findings related to ulnar nerve symptoms at postoperative year 1. CM joint, carpometacarpal joint; MMT, manual muscle testing; FDP, flexor digitorum profundus; ADQ, abductor digiti quinti; FDI, first dorsal interosseous; SW test, Semmes–Weinstein test; MCV, motor conduction velocity.

Items	Findings
Tenderness points	Proximal to the Guyon’s canal, medial epicondyle of the humerus, and medial intermuscular septum of the upper arm
Physical test	Elbow flexion test (+); Froment test (na: due to CM joint osteoarthritis)
MMT (right/ left)	FDP (ring and little fingers): 5/5 ADQ: 4/5 FDI: 5/5 lumbricals (3rd, 4th): 4/5
SW test (palm)	all green
Terminal latency of ulnar nerve (right/left))	2.5/ 2.5 msec
MCV of ulnar nerve	Below elbow: 68.5 / 65.4 m/s Above elbow: 59.5 / 74.7 m/s
QuickDASH score (disability and pain)	25

POY 1.5

Numbness in the ulnar nerve distribution persisted, whereas the muscle strength of the ADQ and the 3rd and 4th lumbricals had recovered to MMT grade 5. Nighttime use of an elbow extension splint relieved morning symptoms.

POY 2

The patient remained stable without worsening of numbness.

POY 2.5

Tenderness and Tinel-like sign at the Guyon’s canal resolved, whereas those at the cubital tunnel remained. The intensity of numbness in the palmar and dorsal aspects became comparable.

POY 3.5

Numbness in the ulnar nerve distribution remained tolerable and stable over the long term. The patient declined further surgery and chose to continue long-term follow-up.

## Discussion

In this case, a lipoma arising from the flexor tenosynovium simultaneously compressed the median nerve and the DCBUN. Lipomas of the hand are uncommon, representing approximately 1-3.8% of all lipomas [8], and cases associated with compressive neuropathy are even rarer. Only two cases have been reported in which a lipoma arising from the flexor tenosynovium involved either the median or the ulnar nerve [9,10]. Furthermore, five cases have described simultaneous compression of both nerves by a single lipoma [11]. In those previous reports, the lipomas responsible for carpal and Guyon’s canal syndromes were large, which made the diagnosis straightforward [9-11]. In contrast, the present case was characterized by a small, deep-seated lipoma that caused simultaneous entrapment of the median and ulnar nerves. Such deep and clinically unapparent lipomas are often referred to as “occult lipomas [12].” In the present case, the occult lipoma was incidentally detected on MRI performed to evaluate the enlarged median nerve. MRI is useful in evaluating CTS to differentiate space-occupying lesions, osseous or articular disorders, and neoplastic conditions as possible underlying causes. Previous studies have reported that ganglion cysts, Kienböck’s disease, and synovitis are among the most frequently detected findings [13]. Marked enlargement of the median nerve warrants consideration of lipofibromatous hamartoma and peripheral nerve sheath tumors such as neurofibroma and schwannoma [14,15]. In atypical cases of CTS, such as those with early onset, unilateral involvement, subcutaneous mass, or marked nerve enlargement, MRI should be performed to aid in differential diagnosis [13].

A diagnostic challenge in the present case was the delayed recognition of DCBUN involvement due to anchoring bias. Although the patient had complained of ulnar-sided symptoms from the first visit, clinical attention was drawn to the more apparent findings of CTS. Consequently, the ulnar involvement was not fully recognized until one year after surgery. Additionally, because our institution does not routinely perform sensory nerve action potential testing, DCBUN involvement could not be diagnosed electrophysiologically. As a result, the lipoma surrounding the DCBUN was not removed during the initial surgery, which made differentiation from the subsequently developed cubital tunnel syndrome more complex. In retrospect, it would have been preferable to excise the tumor around the DCBUN simultaneously during the initial surgery. This case highlights that even a relatively small occult lipoma can cause multiple nerve symptoms, underscoring the importance of carefully evaluating patients’ subjective complaints to avoid diagnostic oversight.

## Conclusions

We reported a rare case in which a small lipoma arising from the flexor tenosynovium caused simultaneous CTS and DCBUN neuropathy. MRI was useful for identifying the lesion; however, anchoring bias toward CTS resulted in delayed recognition of the DCBUN involvement. Because DCBUN neuropathy presents with few objective findings, diagnosis can be challenging. Clinicians should be aware that even a relatively small occult lipoma can compress multiple nerves, emphasizing the importance of careful assessment of patients’ symptoms and the early use of MRI when indicated.
